# Potential of *Oryza officinalis* to augment the cold tolerance genetic mechanisms of *Oryza sativa* by network complementation

**DOI:** 10.1038/s41598-018-34608-z

**Published:** 2018-11-05

**Authors:** Ai Kitazumi, Isaiah C. M. Pabuayon, Hajime Ohyanagi, Masahiro Fujita, Bipush Osti, Matthew R. Shenton, Yusuke Kakei, Yasukazu Nakamura, Darshan S. Brar, Nori Kurata, Benildo G. de los Reyes

**Affiliations:** 1 Department of Plant and Soil Science, 219 Experimental Sciences Building, Texas Tech University, Lubbock, TX 79409 USA; 2 Plant Genetics Laboratory, National Institute of Genetics, Mishima, Shizuoka, 411-8540 Japan; 3 King Abdullah University of Science and Technology (KAUST), Computational Bioscience Research Center (CBRC), Thuwal, 23955-6900 Saudi Arabia; 4 School of Agriculture, Meiji University, 1-1-1 Higashi-Mita, Tama-ku, Kawasaki-shi, Kanagawa 214-8571 Japan; 5 Institute of Vegetable and Floriculture Science, National Agriculture and Food Research Organization, Mie, 514-2392 Japan; 6 Genome Informatics Laboratory, National Institute of Genetics, 1111 Yata, Mishima, Shizuoka, 411-8540 Japan; 7 Plant Breeding, Genetics and Biotechnology Division, International Rice Research Institute, Los Banos, Philippines

## Abstract

*Oryza officinalis* is an accessible alien donor for genetic improvement of rice. Comparison across a representative panel of *Oryza* species showed that the wild *O*. *officinalis* and cultivated *O*. *sativa* ssp. japonica have similar cold tolerance potentials. The possibility that either distinct or similar genetic mechanisms are involved in the low temperature responses of each species was addressed by comparing their transcriptional networks. General similarities were supported by shared transcriptomic signatures indicative of equivalent metabolic, hormonal, and defense status. However, *O*. *officinalis* has maintained an elaborate cold-responsive brassinosteroid-regulated *BES1*-network that appeared to have been fragmented in *O*. *sativa*. *BES1*-network is potentially important for integrating growth-related responses with physiological adjustments and defenses through the protection of photosynthetic machinery and maintenance of stomatal aperture, oxidative defenses, and osmotic adjustment. Equivalent physiological processes are functional in *O*. *sativa* but their genetic mechanisms are under the direct control of ABA-dependent, DREB-dependent and/or oxidative-mediated networks uncoupled to *BES1*. While *O*. *officinalis* and *O*. *sativa* represent long periods of speciation and domestication, their comparable cold tolerance potentials involve equivalent physiological processes but distinct genetic networks. *BES1*-network represents a novel attribute of *O*. *officinalis* with potential applications in diversifying or complementing other mechanisms in the cultivated germplasm.

## Introduction

The domesticated rice (*Oryza sativa* L.) represents only a fraction of the total genetic potential of the genus^1–3^. Geographic distribution, developmental and physiological variation across cultivars and wild species reflect a gradient of ecological adaptation that were optimized during speciation and domestication^4^. Taxonomic sub-division in the genus includes the *sativa*, *officinalis*, *ridleyi*, and *granulata* species complexes, and sections *ridleyanae Tateoka* and *brachyantha B*.*R*. *Lu*. Under these sub-divisions are two cultivated and 22 wild species, defined by six diploid (2n = 24; AA, BB, CC, EE, FF, GG) and four allotetraploid (4n = 48; BBCC, CCDD, HHJJ, KKLL) genomes^5,6^. The *sativa*-complex includes the temperate (*japonica*) and tropical (*indica*) Asian rice *O*. *sativa*, African rice *O*. *glaberrima*, and their AA-genome progenitors *O*. *rufipogon*, *O*. *nivara*, and *O*. *barthii*, respectively. Harnessing the potential of this gene pool to widen the genetic base of cultivars has been successful^2^.

The *officinalis*-*complex* is the largest group that includes the genomes BB (*O*. *punctata*), CC (*O*. *officinalis*, *O*. *rhizomatis*, *O*. *eichingeri*) and EE (*O*. *australiensis*), and their allotetraploid combinations BBCC (*O*. *minuta*, *O*. *punctata*) and CCDD (*O*. *latifolia*, *O*. *alta*, *O*. *grandiglumis*). *O*. *officinalis* is a highly valued member of this complex because of its pivotal role in multiple tetraploidization events^7^. Habitat distribution indicate that *O*. *officinalis* is a rich reservoir of adaptive traits for the enhancement of cultivars^8–12^. Successful efforts for agronomic trait introgression from wild *Oryza* to cultivars involved either a diploid CC-genome *(O*. *officinalis*) or allotetraploid CC-combination (*O*. *minuta*, *O*. *latifolia*) as donors^13–15^. This shows that CC-genome is a more accessible alien donor for the diversification of the genetic base of cultivars^16^.

While the Asian cultivated rice is generally very cold-sensitive, a number of temperate japonica cultivars have been used as donors of tolerance-associated traits^17,18^. Based on population distribution and eco-geographic niches, bioclimatic models placed *O*. *officinalis* in the middle range of stress tolerance potentials relative to 21 other *Oryza* species^4^. However, despite the predictions of the bioclimatic models, the true potentials of the wild *Oryza* germplasm as a source of novel mechanisms for cold tolerance that may not exist in temperate japonica have not been critically examined.

In this study, the cold stress transcriptional network of the CC-genome reference *O*. *officinalis* accession IRGC100896 was reconstructed and compared with the corresponding network in the AA-genome reference *O*. *sativa* ssp. japonica cv. Nipponbare. The IRGC100896 was chosen as reference *O*. *officinalis* because it is widely used as an alien donor in rice breeding^16,19^. The major goal was to reveal shared and contrasting regulatory network signatures across the two reference genotypes with comparable cold tolerance potentials in order to understand the significance of such networks in context of possible complementation effects in recombinants. The genetic mechanism revealed in this study is an important first step for understanding the finer details behind the hidden potentials of the *Oryza* CC-genome for diversifying the genetic mechanisms that exist in cultivars.

## Results

### Cold tolerance potential of IRGC100896 and Nipponbare

**Figure 1:**
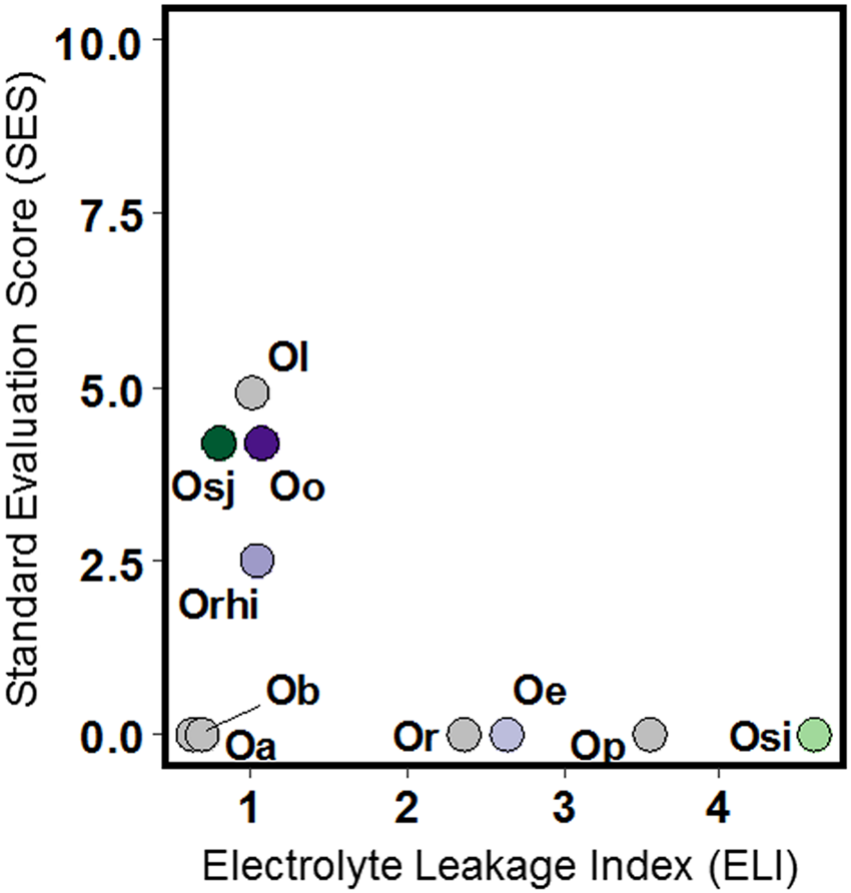
Comparison of plant injury by tissue electrolyte leakage index (ELI) and plant recovery expressed as standard evaluation score (SES) across a panel of wild and cultivated *Oryza*. Recovery is expressed in a scale of 0 (0%) to 10 (100%). ELI is the ratio of induced electrolyte leakage between control and stress conditions, with ELI >1.0 indicating significant injury (p < 0.05, n = 6). *Cultivars*: Osj = *sativa-japonica*, Nipponbare; Osi = *sativa-indica*, IR64; *AA-genome*: Or = *rufipogon*, Ol = *longistaminata*; *CC-genome*: Oo = *officinalis*; Oe = *O*. *eichingeri*; Or = *rhizomatis*; *EE-genome*: Oa = *australiensis*; *FF-genome*: Ob = *brachyantha*; *CCDD-genome*: Op = *punctata*.

Guided by the bioclimatic models of Atwell *et al*.^6^, a panel of wild accessions used as alien donors in IRRI’s breeding program was compared for sensitivity to low temperature (4 °C). Evaluation was based on standardized metrics that included IRRI’s Standard Evaluation Score (SES) for plant recovery, and plant injury measurements through the cellular electrolyte leakage index (ELI)^18^. Overall, the relative ranking of accessions revealed by SES and ELI was generally consistent with the proposed ranking in the bioclimatic model. The IRGC100896 and Nipponbare were more similar to each other in terms of cold tolerance, both having significantly lower ELI and SES compared to the highly sensitive check IR64 (*O*. *sativa* ssp. indica) and other diploid wild accessions (Fig. 1; Supplementary Figure S1).

### General features of CC and AA cold stress transcriptomes

**Figure 2:**
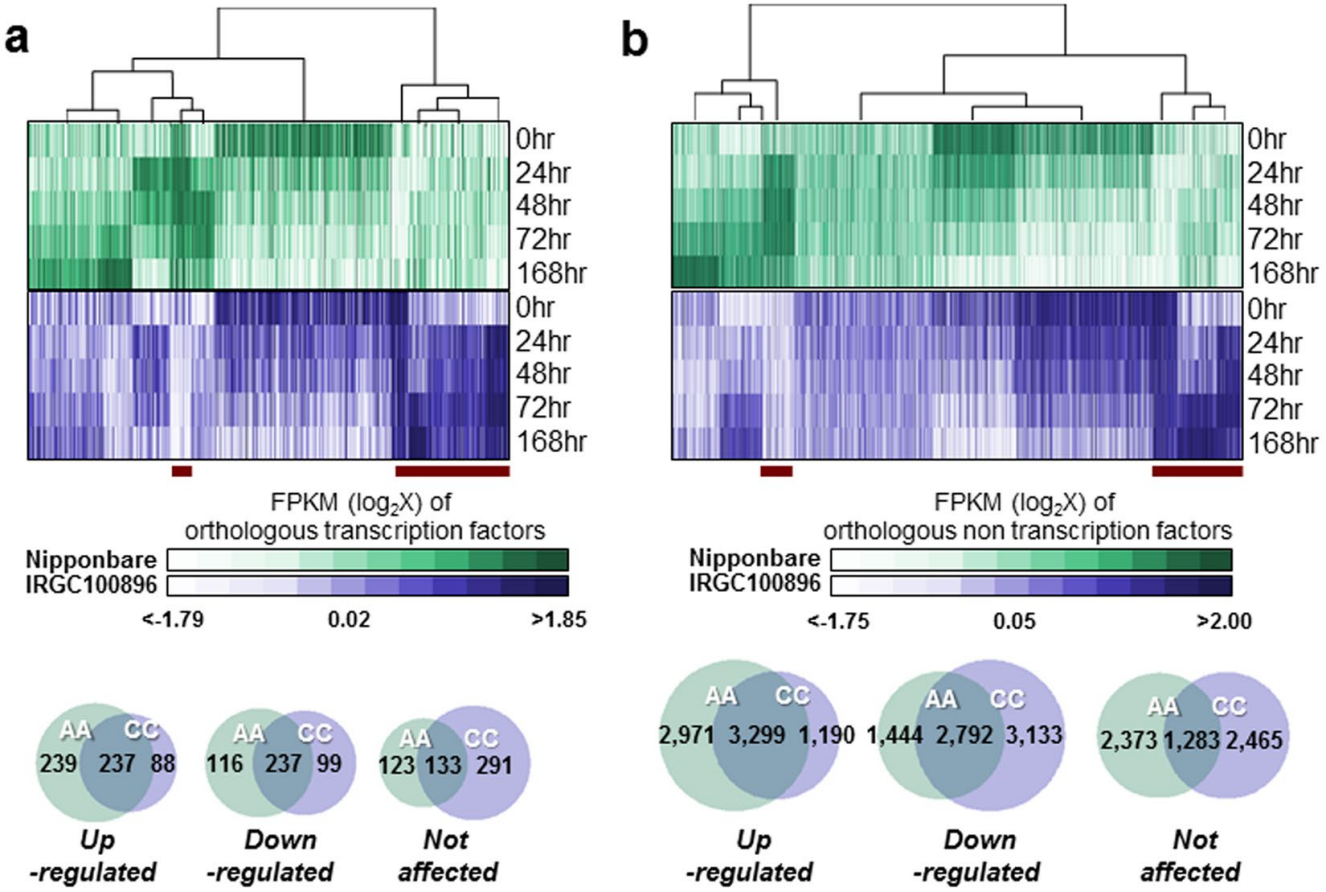
General trends in the cold stress transcriptomes of *O*. *officinalis* (IRGC100896; purple) and *O*. *sativa* ssp. japonica (Nipponbare; green). Mapped reads in each RNA-Seq library were normalized to allow direct comparison of relative transcript abundance across orthologous loci. (**a**) Top panel shows the temporal expression of 943 orthologous pairs of transcription factors in IRGC100896 and Nipponbare. Profiles that are common between the two genotypes and those unique to one genotype (red bars) are indicated. Venn diagram (bottom) summarizes the proportion of orthologous transcription factors with either similar or unique profile across genotypes. (**b**) Expression patterns across 14,162 pairs of orthologous non-transcription factors in IRGC100896 and Nipponbare presented in similar context as in (**a**).

A series of RNA-Seq libraries (*DDBJ-DRA006704*) was constructed to investigate whether distinct mechanisms are involved in the expression of similar cold tolerance potential in IRGC100896 and Nipponbare *(*Supplementary Table S2). RNA-Seq libraries of Nipponbare detected 1,168 unique transcription factor transcripts and 20,699 unique non-transcription factor transcripts across expression profiles, which represent 88% and 87% of the total transcripts for each category in the Nipponbare reference. These results are generally consistent with previously reported microarray-based transcriptome datasets^20–22^. RNA-Seq libraries of IRGC100896 detected 1,107 unique transcription factor transcripts and 17,403 unique non-transcription factor transcripts across expression profiles, representing 83% and 73% of total transcripts for each category in the reference (Fig. 2*;* Supplementary Figure S3).

Overall, expression of orthologous genes did not follow a perfect one-to-one trend across the IRGC100896 and Nipponbare transcriptomes, which indicated that both overlapping and unique sets of genes are involved in each genotype’s genetic networks (Fig. 2*;* Supplementary Figure S3). Among the transcripts expressed from orthologous genes, 476, 211, and 256 represent transcription factors that were upregulated, downregulated, and not affected by cold, respectively in Nipponbare. In IRGC100896, 325, 194, and 424 transcription factors were upregulated, downregulated and not affected by cold, respectively. Only 50% of cold-upregulated transcription factors in Nipponbare were also upregulated in IRGC100896, while only 73% of cold-upregulated transcription factors in IRGC100896 were also upregulated in Nipponbare. Similar reciprocal and non-reciprocal patterns were observed for downregulated genes.

### Metabolic status inferred from cold stress transcriptome signatures

**Figure 3:**
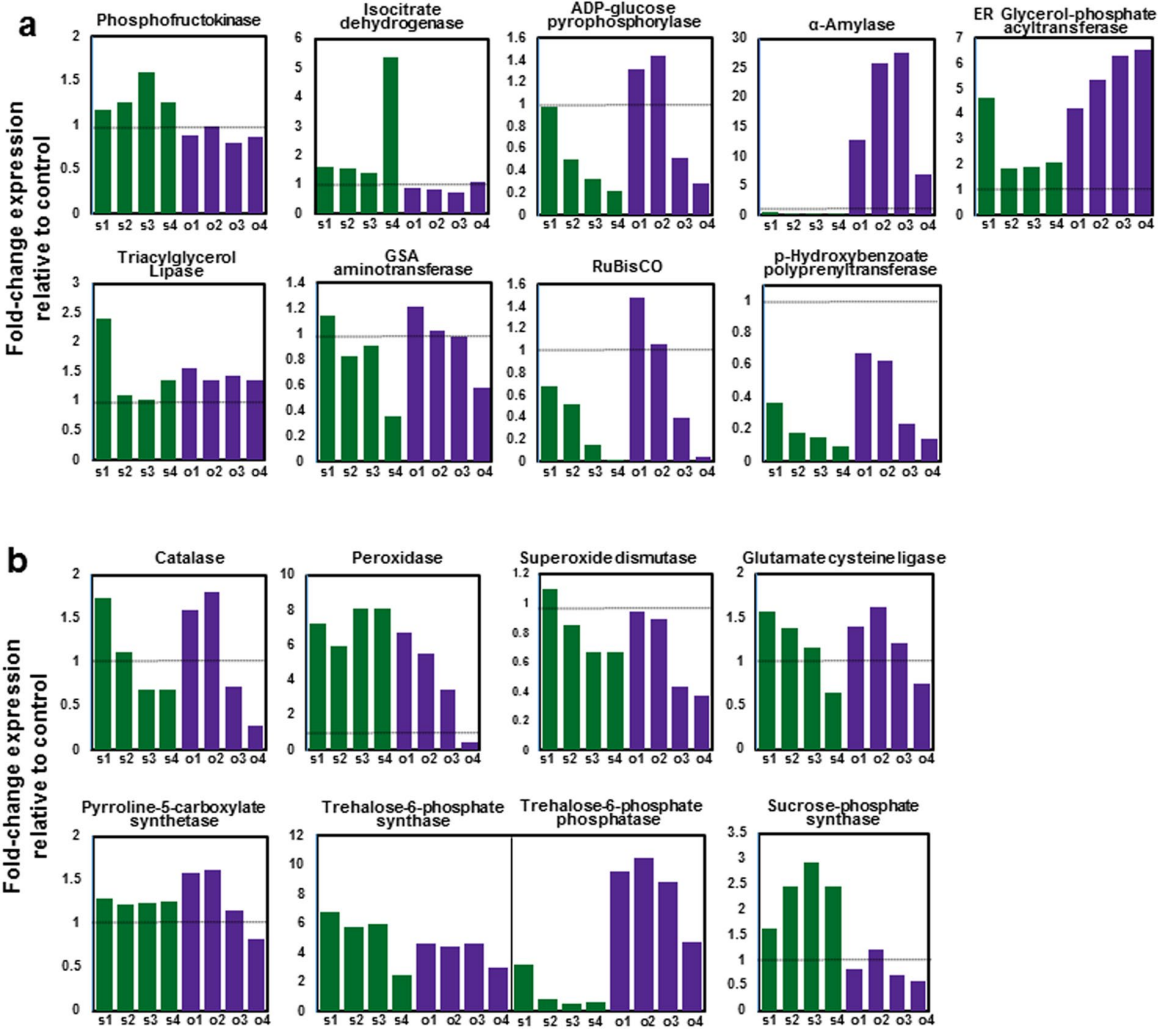
Transcriptome signatures representing the primary metabolic, radical scavenging, and osmotic adjustment status of IRGC100896 (*purple*) and Nipponbare (*green*). The *s1/o1*, *s2/o2*, *s3/o3*, and *s4/o4* notations represent the transcript fold-change in Nipponbare (*s*) and IRGC100896 (*o*) at 24, 48, 72 and 144 hr, respectively. (**a**) Expression of rate-limiting enzymes *phosphofructokinase* (glycolysis), *isocitrate dehydrogenase* (TCA cycle), *ADP-glucose phosphorylase* (starch biosynthesis), α*-amylase* (starch catabolism), *glycerol-phosphate acyltransferase* (triacylglyceride biosynthesis), *triacylglycerol lipase* (triacylglyceride catabolism), *GSA aminotransferase* (chlorophyll biosynthesis), *RuBisCO* (photosynthesis/Calvin cycle), and *p-hydroxybenzoate polyprenyltransferase* (ubiquinone biosynthesis). KaPPA-View transcript maps in Supplementary Figures S4–S10. (**b**) Differential expression of radical scavenging (*catalase*, *peroxidase*, *superoxide dismutase*, *glutathione synthesis*), trehalose biosynthetic (*trehalose-6-phosphate synthase*, *trehalose-6-phospate phosphatase*), proline biosynthetic (*pyrroline-5-carboxylate synthetase*), and sucrose biosynthetic (*sucrose-phosphate synthase*) genes in IRGC100896 (*purple*) and Nipponbare (*green*). KaPPA-View maps are in Supplementary Figures S11–S12.

To compare the physiological status of IRGC100896 and Nipponbare under cold stress, KaPPA-View transcript maps were assembled for the primary metabolic pathways (Fig. 3a*;* Supplementary Figures S4–S8). Glycolysis and TCA cycle were not drastically different across genotypes based on the expression of their respective rate-limiting enzymes *phosphofructokinase* (EC.2.7.1.90) and *isocitrate dehydrogenase* (EC.1.1.1.42). Starch biosynthetic and catabolic pathways were assessed through the expression of *ADP-glucose pyrophosphorylase* (E.C.2.7.7.27) and α-*Amylase* (E.C.3.2.1.1), respectively. Opposite trends for biosynthesis and catabolism suggest less perturbation or penalty in IRGC100896. Based on the expression of *glycerol-phosphate acyltransferase* (E.C.2.3.1.15), triacylglyceride biosynthesis appeared to be differentially affected by cold, being highly upregulated in IRGC100896. Triacylglyceride catabolism as reported by *triacylglycerol lipase* (E.C.3.1.1.3) expression was not very different. Based on the expression of the enzyme *p-hydroxybenzoate polyprenyltransferase* (E.C.2.5.1.39) for ubiquinone and ATP biosynthesis, it appeared that the energy status of IRGC100896 and Nipponbare were similarly impaired under cold stress. As expected, photosynthesis was generally impaired in both genotypes as indicated by the downregulation of the rate-limiting biosynthetic enzyme *glutamate-1-semialdehyde aminotransferase* (E.C.5.4.3.8). Reduced activity of Calvin cycle as suggested by downregulation of RuBisCo (E.C.4.1.1.39) was consistent with the downregulation of chlorophyll biosynthesis (Fig. 3a*;* Supplementary Figures S9, S10).

Primary defense capacity by radical scavenging (peroxidases, catalases, superoxide dismutases, glutathione-SH), and osmotic adjustment (sucrose, trehalose, proline) were also compared (Fig. 3b*;* Supplementary Figures S11, S12). *Catalase* (E.C.1.11.1.6; Os03g0131200) and *peroxidase* (E.C.1.11.1.7; Os02g0240300) were upregulated in both genotypes, particularly during the early periods of stress, while *superoxide dismutase* (E.C.1.15.1.1; Os05g0323900) was not drastically affected. Glutathione biosynthesis as indicated by the expression of *glutamate cysteine ligas*e (E.C.6.3.2.2; Os07g0462000, Os05g0129000) was similar to the patterns of *catalase* and *peroxidase*, suggesting that the mechanisms of radical scavenging are similar in IRGC100896 and Nipponbare. Trehalose biosynthesis as indicated by the expression of *trehalose-6-phosphate synthase* (E.C.2.4.1.15) and *trehalose-6-phosphate phosphatase* (E.C.3.1.3.12) appeared to be higher IRGC100896. Increased expression of *pyrroline-5-carboxylate synthetase* (E.C.2.7.2.11 1.2.1.41; Os05g0455500) suggests an enhanced proline biosynthesis in both genotypes. Sucrose biosynthesis as indicated by *sucrose-phosphate synthase* (E.C.2.7.2.11; E.C.1.2.1.41) was upregulated in Nipponbare but downregulated in IRGC100896.

### Hormonal status inferred from transcriptome signatures

**Figure 4:**
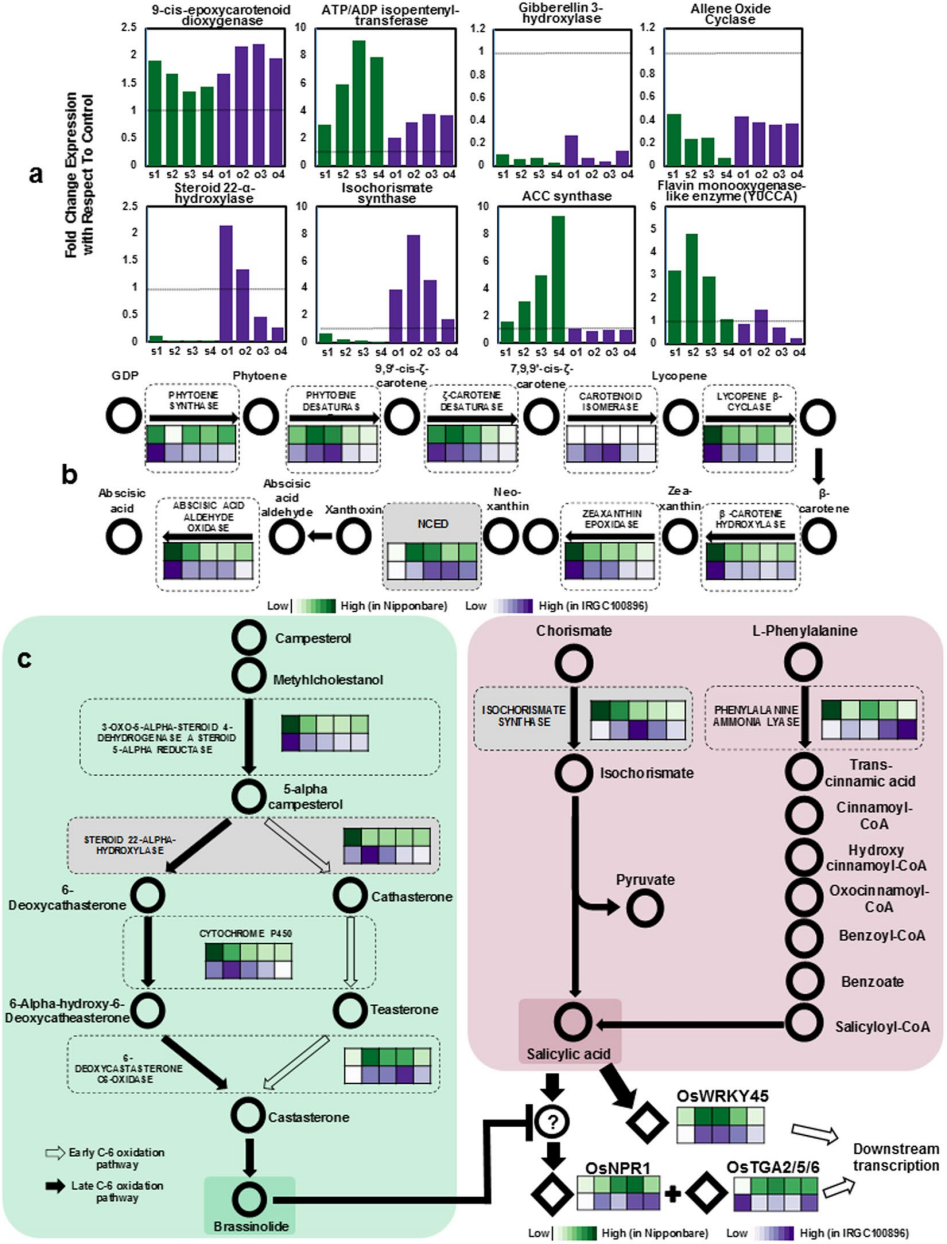
Transcriptome signatures representing the hormone biosynthetic status in IRGC100896 (*purple*) and Nipponbare (*green*). (**a**) Expression of rate-limiting enzymes *9-cis-epoxycarotenoid dioxygenase* or *NCED* (ABA), *ATP/ADP isopentenyl transferase* (ZT biosynthesis), *gibberellin 3-hydroxylase* (GA) and *allene oxide cyclase* (JA), *steroid 22-*α*-hydroxylase* (BL), *isochorismate synthase* (SA), *ACC synthase* (C_2_H_4_) and *tryptamine monooxygenase* (IAA). The *s1/o1*, *s2/o2*, *s3/o3*, and *s4/o4* notations represent the transcript fold-change in Nipponbare (*s*) and IRGC100896 (*o*) at 24, 48, 72 and 144 hr, respectively. (**b**) KaPPA-View transcript maps showing upregulation of ABA biosynthesis in both IRGC100896 (*purple*) and Nipponbare (*green*). (**c**) Parallel upregulation of BL and SA biosynthesis in IRGC100896 (*purple*) but not in Nipponbare (*green*), and cross-talks between BL and SA pathways through *OsNPR1*, *OsTGA2/5/6* and *OsWRKY45*. Rate-limiting steps are highlighted in the grey box. Maps for other hormones are in Supplementary Figures S13–S17.

Stress and growth-related responses are integrated by hormonal signals. To compare the relative activities of hormone biosynthetic pathways across IRGC100896 and Nipponbare, KaPPA-View pathway maps were established for abscisic acid (ABA), gibberellic acid (GA), auxin (IAA) cytokinin (ZT), brassinosteroid (BL), salicylic acid (SA), ethylene (C_2_H_4_), and jasmonic acid (JA). Patterns in GA, ZT, and JA pathways were very similar across the two genotypes as indicated by the expression of their rate-limiting enzymes (Fig. 4a, b*;* Supplementary Figures S13–S15). Based on the expression of *9-cis-epoxycarotenoid dioxygenase* (*NCED*; EC 1.13.11.51) and *ATP/ADP isopentenyl transferase* (E.C.2.5.1.8), ABA and ZT pathways appeared to be similarly enhanced by cold in both genotypes. GA and JA pathways were similarly downregulated as indicated by rate-limiting enzymes *GA3-hydroxylase* (E.C.1.14.11.15) and *allene oxide cyclase* (E.C.5.3.99.6). Downregulation of these pathways correlate well with the observed impairment of plant growth under cold stress.

BL, SA, C_2_H_4_, and IAA pathways had the most significant contrasts between IRGC100896 and Nipponbare, based on the expression of their rate-limiting enzymes *steroid 22-alpha-hydroxylase* (E.C.1.14.13.-), *isochorismate synthase* (E.C.5.4.4.2), *ACC synthase* (E.C.4.4.1.14), and *YUCCA flavin monooxygenase (*E.C.1.14.13.8), respectively (Fig. 4c*;* Supplementary Figures S16–S17). The most notable contrast was the upregulation of BL and SA pathways in IRGC100896 but downregulation in Nipponbare. C_2_H_4_ and IAA pathways were upregulated in Nipponbare but downregulated in IRGC100896, consistent with the observed senescence and differential patterns in chlorophyll biosynthesis and Rubisco activity.

### Transcriptional consequences of BL biosynthetic signatures

Analysis of the upstream sequences of genes that were cold-responsive in IRGC100896 identified the brassinosteroid response elements (*BRRE)* among the most significantly enriched motifs (Supplementary Figure S18). To establish a network of genes associated with the enhanced BL biosynthesis in IRGC100896, transcription factors that were cold-upregulated in IRGC100896 but not in Nipponbare were identified. It was assumed that such group of transcription factors are likely to include the direct targets of enhanced BL biosynthesis in IRGC100896, and their transcriptional networks can be reconstructed by capturing other co-expressed genes.

**Figure 5:**
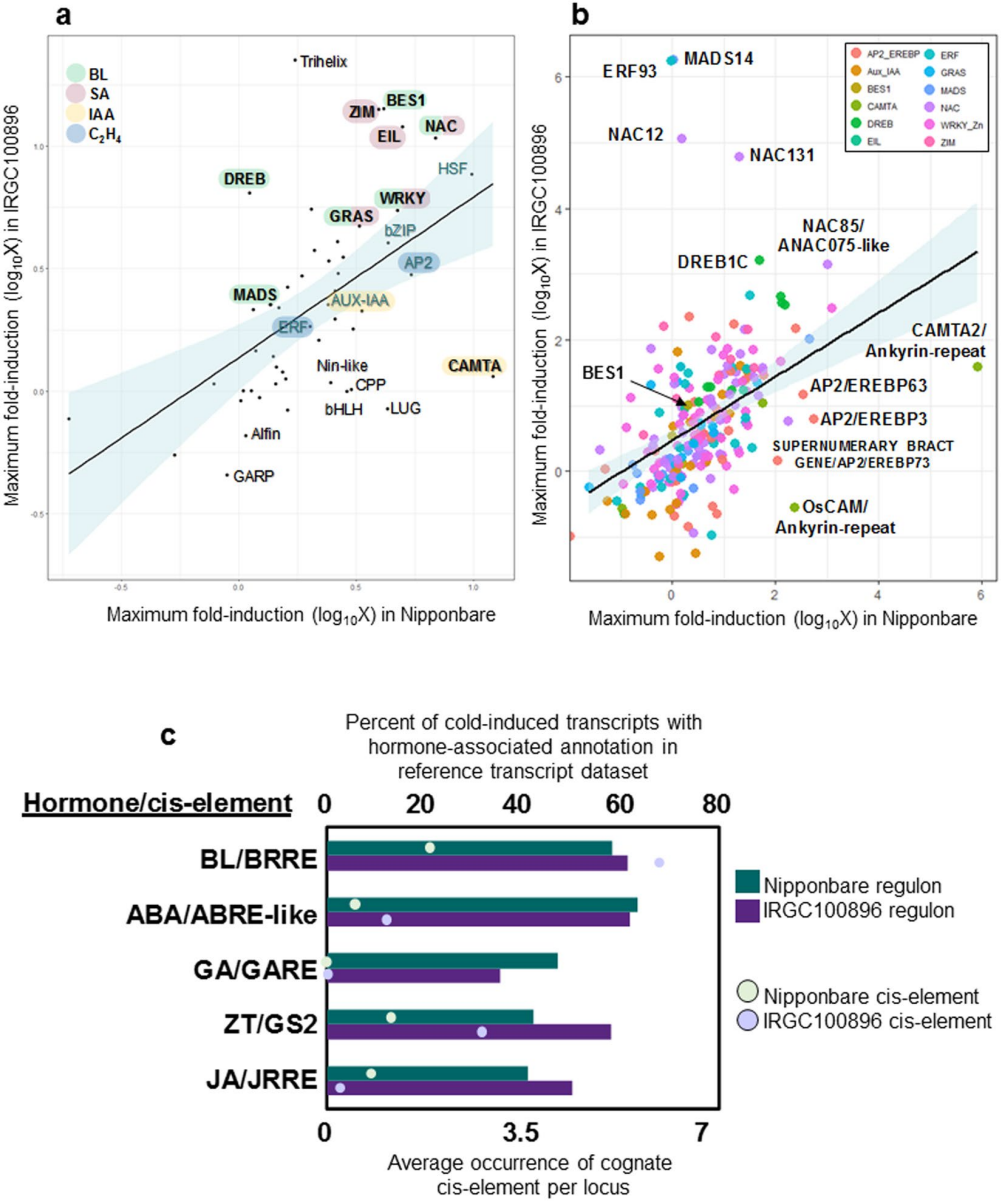
Shared or unique network signatures across IRGC100896 and Nipponbare. (**a**) Transcription factor families that were similarly or uniquely expressed across IRGC100896 and Nipponbare. *BES1*, *NAC*, *WRKY*, *DREB*, *GRAS a*nd *MADS* are BL-associated transcription factors that were upregulated in IRGC100896 but not in Nipponbare. *WRKY*, *NAC* and ZIM are involved in cross-talks between BL and SA that were upregulated in IRGC100896 but not in Nipponbare. (**b**) Non-differentially expressed and differentially expressed transcription factors across IRGC100896 and Nipponbare. (**c**) Cold-upregulated genes associated hormone response in RiceXPro were examined for *BRRE (BL*), *ABRE-like (ABA*), *GARE (GA*), *GS2 (ZT*), and *JRRE (JA*) enrichment along the −1,500 to +500 regions. Enrichment of each class of cis-elements within a cluster of co-regulated genes are shown by the colored dots across the bar graph.

Patterns of transcription factor expression are shown in Fig. 5a and b with expression in Nipponbare in x-axis and IRGC100896 in y-axis (Supplementary Figure S19). Upregulation of bZIP transcription factors associated with ABA-responses is an example of a common signature of IRGC100896 and Nipponbare, mirroring the enhanced expression of ABA biosynthetic genes in both genotypes (Fig. 4*;* Supplementary Figures S13–S15). On the other hand, transcription factors that were most prominently upregulated only in IRGC100896 include *BES1* (Os07g0580500), *NAC* (Os12g0123700), *WRKY* (Os05g0322900), and *DREB2B* (Os02g0752800) (Fig. 5a,b). *BES1* is a well-known regulator of BL-mediated transcription, while *NAC*, *WRKY*, and *DREB2B* are known downstream targets of *BES1*. BL signaling is known to cross-talk with SA signaling through *NAC*, *EIL*, *WRKY*, *GRAS*, and *ZIM* transcription factors, all of which were upregulated in IRGC100896. Upregulation of these transcription factors was consistent with parallel upregulation of genes in the BL and SA biosynthetic pathways. Transcription factors associated with IAA (*Aux/IAA*, *CAMTA)*, and C_2_H_4_ *(AP2/ERF*, *bHLH*) signaling were upregulated only in Nipponbare, mirroring the general trends in IAA and C_2_H_4_ biosynthetic pathways (Fig. 4*;* Supplementary Figures S13–S15).

To establish a network of BL-regulated genes in IRGC100896, the cold-induced portion of the transcriptome was further searched for genes with BL-associated functional annotation. The enrichment of cis-elements associated with known hormone-responsive genes in Nipponbare was also investigated across each co-expressed cluster (Fig. 5c). While the number of annotated BL-regulated genes that were also induced by cold was very similar in IRGC100896 and Nipponbare, the IRGC100896 cluster was more significantly enriched with brassinosteroid-response elements (*BRRE*). Co-regulated clusters for ABA, GA, CYT, and JA, which were very similar in composition in IRGC100896 and Nipponbare, also had very similar enrichments of associated cis-elements *ABRE*, GARE, AuxRE, GS2, JRRE, respectively. These trends suggest that the cold stress genetic networks mediated by ABA, GA, CYT, and JA are shared features of IRGC100896 and Nipponbare, while the genetic networks mediated by BL with *BES1* as its core, and its possible interaction with SA signaling represents a unique feature of IRGC100896.

**Figure 6:**
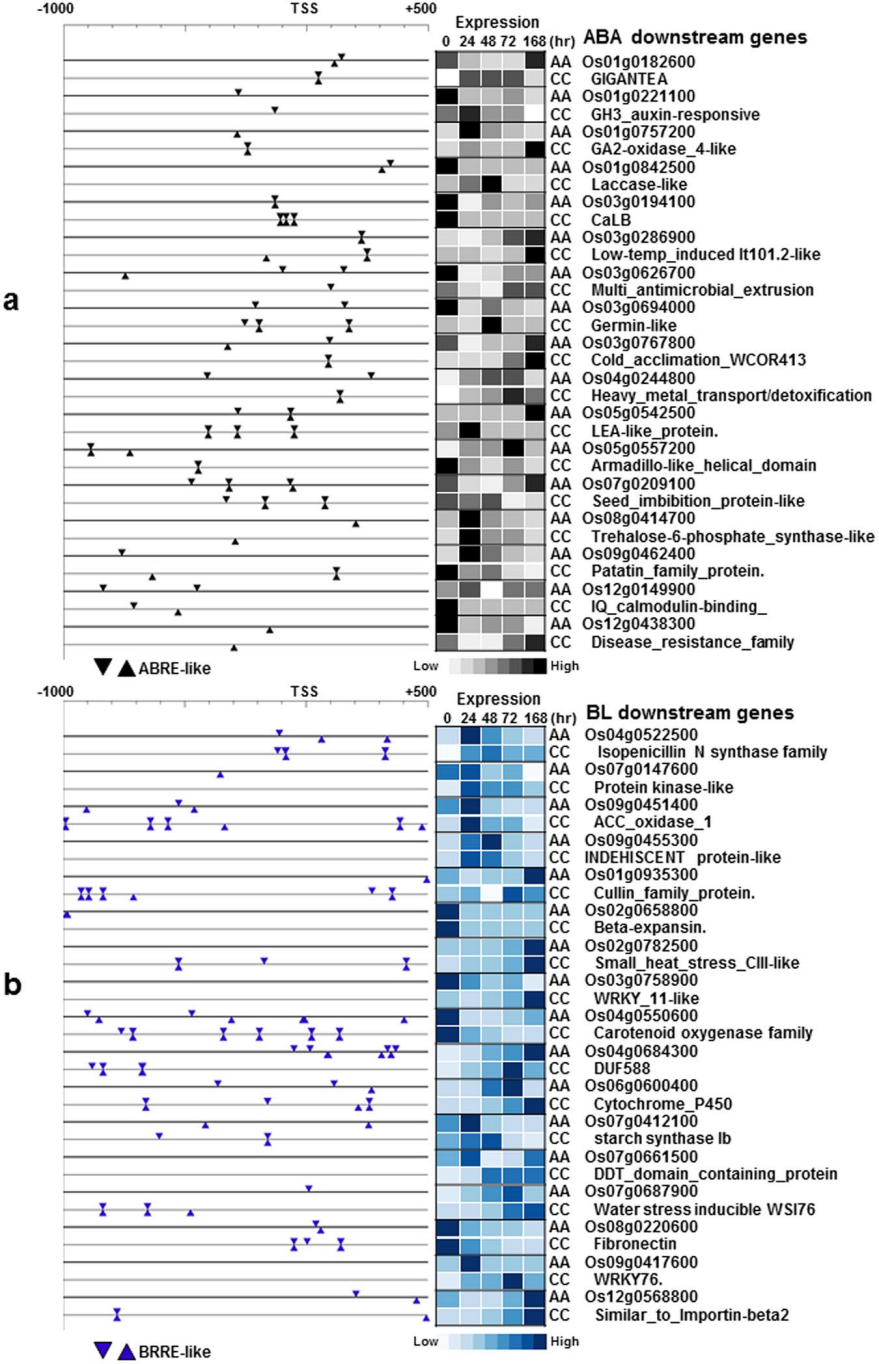
Distribution of critical cis-element motifs (−1,000 to +500) among genes associated with ABA-network (**a**) and those associated with *BES1* network (**b**). Cis-element maps show similar enrichment of *ABRE*-like motifs across the co-regulated clusters in both IRGC100896 and Nipponbare. Cis-element maps for *BRRE* show an enrichment bias towards the *BES1*-associated genes in IRGC100896.

Figure 6 shows the gene-by-gene analysis of cis-element spatial distribution across a subset of co-regulated genes in the ABA network that is common between IRGC100896 and Nipponbare, and BL network that is unique to IRGC100896. Patterns of cis-element enrichment across these contrasting networks were consistent with the trends in Fig. 5c, suggesting that BL-regulated gene expression is an important feature of *O*. *officinalis* genetic mechanism that is deemphasized in *O*. *sativa* ssp. japonica.

### Cold stress mediated BES1-network

The *BES1*-network was assembled to facilitate the interpretation of the biological significance of the major contrasts in the genetic mechanisms of IRGC100896 and Nipponbare. A subset of *BRRE*-enriched genes that were also upregulated by cold in IRGC100896 was established (Supplementary Figure S20). For each transcript, upstream sequences of their genomic loci were extracted from the unpublished draft of *O*. *officinalis W0002* genome and Nipponbare reference. Final dataset included 140 pairs of orthologous genes across *O*. *officinalis* and *O*. *sativa*, with either high (8 to 10) or very high (≥10) densities of *BRRE*/*BRRE*-like motifs (Supplementary Table S21).

**Figure 7:**
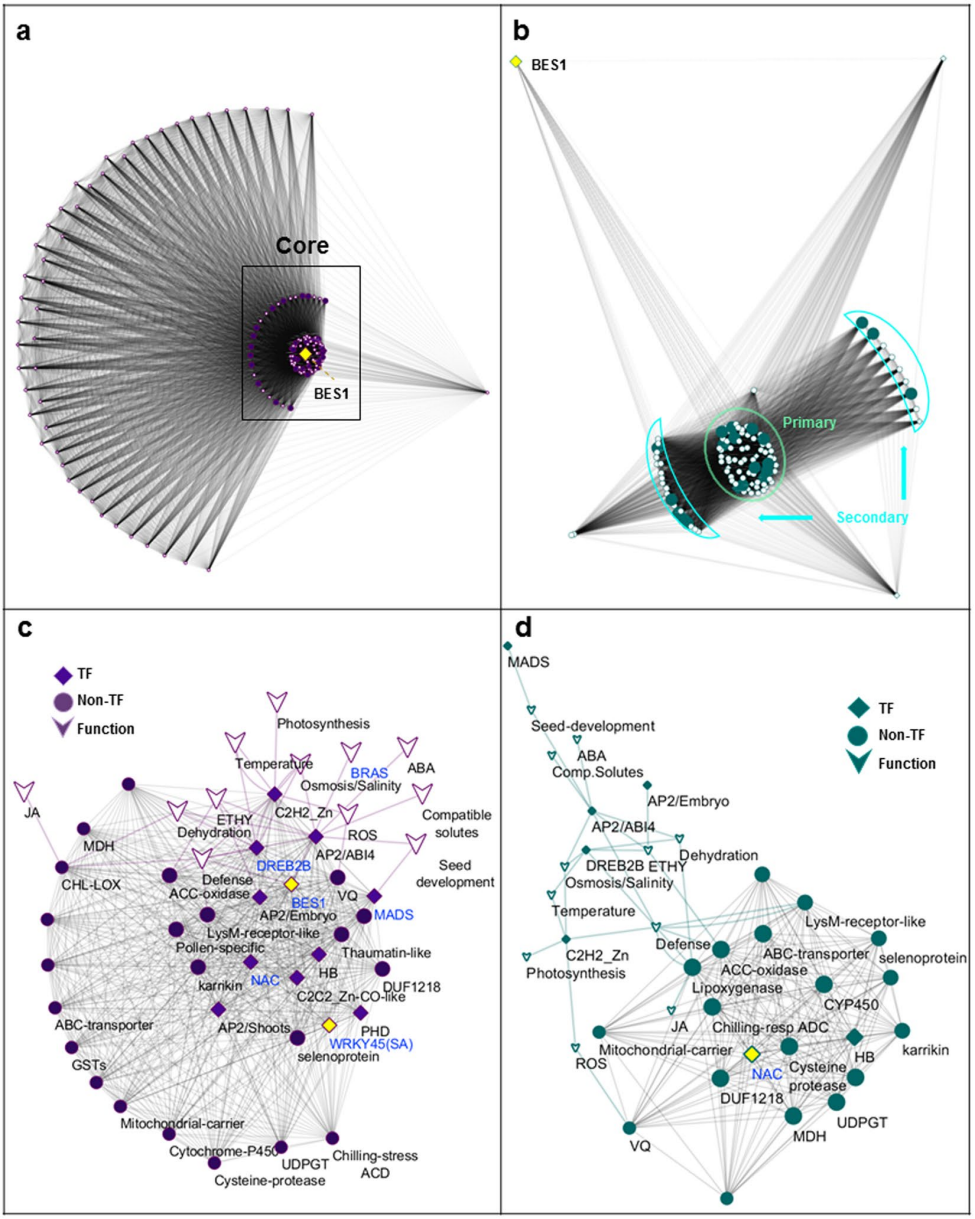
*BES1*-networks in IRGC100896 and Nipponbare based on co-expression and cis-element enrichment. Gene ontology enrichments are shown. (**a**) Larger *BES1*-network in IRGC100896 consisting of 140 most significantly co-expressed genes. Primary, secondary, and tertiary components are shown with *BES1* (*yellow diamond*) at the core. (**b**) Larger network in Nipponbare with linkages among 140 genes that were orthologous to the tightly co-expressed genes in IRGC100896. *BES1* (*yellow diamond*) is not co-expressed with the network components, indicating network fragmentation and uncoupling to BL signaling. (**c**) Primary *BES1-*regulon in IRGC100896 comprised of 34 core network genes, and their associated biological and biochemical functions. Transcription factors downstream to *BES1* (*DERB2B*, *NAC*, *WRKY45*) and associated with SA signaling are shown with direct connection to BL biosynthesis (BRAS). (**d**) Model of *BES1*-independent core regulon consisting of 20 genes that were uncoupled to *BES1* in Nipponbare. The central hub is *NAC* transcription factor downstream to *BES1* in the IRGC100896 core regulon but uncoupled to BL biosynthesis (BRAS).

Figure 7 shows the organization of cold-regulated *BES1*-network in IRGC100896 and Nipponbare, and their putative biochemical and biological functions. The 140-pairs of orthologous genes in the network were all upregulated by cold in IRGC100896 but not necessarily in Nipponbare. In the larger network for IRGC100896 that included all 140 genes (Fig. 7a), *BES1* occupies the core, consistent with the fact that all 140 genes were significantly enriched with *BRRE*. The primary, secondary and tertiary co-expressed genes assembled in an organized fashion around the putative master regulator *BES1*. In contrast, the larger network consisting of all 140 orthologs in Nipponbare appeared fragmented into three sub-networks that were at best only loosely linked to the central hub *BES1 (*Fig. 7b*)*. Patterns in the larger network of Nipponbare were consistent with the fact that *BRRE* was significantly depleted among those 140 genes.

Zoomed-in views of IRGC100896 and Nipponbare networks highlighting the core regulons are shown in Fig. 7c,d. In IRGC100896, the core regulon is comprised of a much larger subset of 34 tightly co-expressed genes that are directly connected to and organized around *BES1*. The significance of this core network is further strengthened by the fact that known *BES1*-target genes (*DREB2B/Os02g0752800*, *NAC/Os12g0123700*, *WRKY45/Os05g0322900*, and *MADS-box/Os02g0170300*) were indeed tightly co-expressed with *BES1*, and captured within its core regulon. These genes are also known regulators of SA signaling, consistent with the observed parallel upregulation of BL and SA biosynthetic pathways.

The biochemical and biological functions associated with the core *BES1*-regulon in IRGC100896 included brassinosteroid biosynthesis (BRAS), responses to dehydration, salinity and cold, responses to ABA, regulation of embryo maturation, seed dormancy, and germination, radical scavenging and oxidative defenses, osmotic adjustment, transport regulation, responses to pathogens, and photosynthesis (Fig. 7c*;* Supplementary Table S21). These functions are directly relevant to physiological adjustments and defenses^21,22^.

The core regulon of Nipponbare was a stark contrast to the core regulon of IRGC100896 for two reasons. First, it was comprised of much fewer genes (total = 20) that formed a relatively dispersed network uncoupled to *BES1* but directly linked to *NAC* transcription factor (*Os1*2*g0123700*) that is downstream to *BES1* in the IRGC100896 network. Second, other transcription factors downstream to *BES1* in the IRGC100896 network (*DREB2B/Os02g0752800*, *MADS-box protein/Os02g0170300*) were in fact not co-expressed in Nipponbare (Fig. 7d). The putative biochemical and biological functions of the *NAC-*regulated core regulon of Nipponbare include responses to dehydration, salinity, cold, and ABA, regulation of embryo maturation and seed dormancy, radical scavenging and oxidative defenses, osmotic adjustment, transport regulation, responses to pathogens, and photosynthesis. These are generally similar to the predicted outcomes of the more elaborate *BES1*-network in IRGC100896.

**Figure 8:**
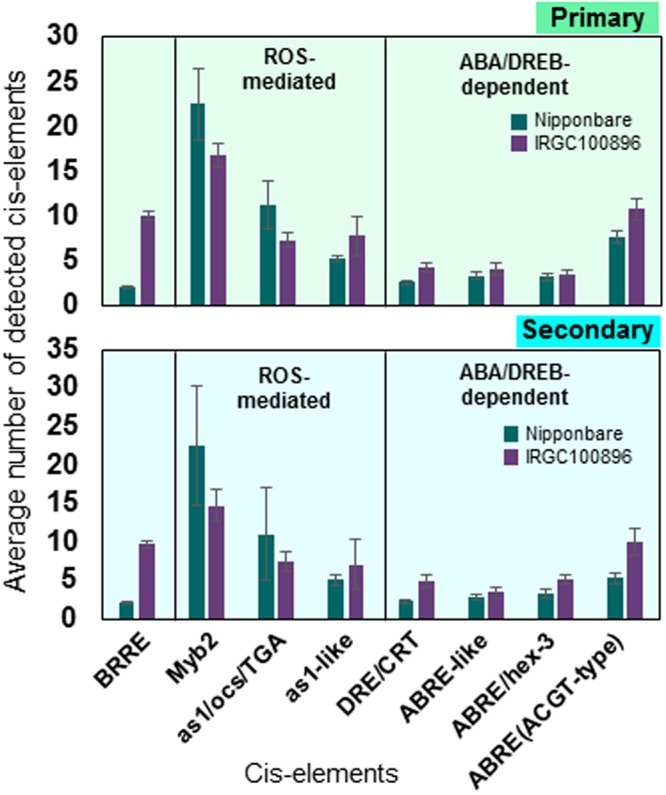
Enrichment of cis-elements associated with ROS-mediated (*Myb2-box*, *as1/ocs/TGA-like*), ABA-dependent (*ABRE*, *ABRE-like*, *ABRE-ACGT*, *hex-3*), and *DREB*-mediated (*DRE/CRT*) regulons across all genes in the *BES1*-network of IRGC100896, and across all genes in *BES1*-independent network of Nipponbare. Patterns of enrichment for these cis-elements across the members of the primary/core and secondary regulons were very similar in IRGC100896 and Nipponbare.

Previous analysis of the Nipponbare cold stress transcriptional network highlighted the primary roles of oxidative-mediated, ABA-dependent, and *DREB*-mediated regulons for early defenses^20–22^. While *BRRE* was a unique signature of cold-regulated genes in IRGC100896, the enrichment of cis-elements associated with ROS-mediated (*Myb2-box*, *as1/ocs/TGA-like*), ABA-dependent (*ABRE*, *ABRE-like*, *ABRE-ACGT*, *hex-3*), and *DREB*-mediated (*DRE/CRT*) regulons were very similar in IRGC100896 and Nipponbare (Fig. 8). These results highlight the importance of *BES1*-network as a defining feature of O. *officinalis* cold stress genetic mechanism.

## Discussion

Sequence variation showed that the CC-genome is more similar to AA-genome than to any other genomes in the genus *Oryza*^5,9,23–29^. Understanding the implications of such variation in context of regulatory networks is important for harnessing novel physiological traits in breeding. Analysis of habitat and geographic distribution in the genus *Oryza* as described in bioclimatic models indicated that temperature and moisture constraints are key determinants of species ecological boundaries. *O*. *officinalis* was placed in the middle of the spectrum of stress tolerance variation^4,24^. Improving cold tolerance has always been a major goal of rice breeding, yet there is no clear consensus as to which *Oryza* species are the most suitable alien genetic donors. The comparability of the cold tolerance potentials of IRGC100896 and Nipponbare^20–22^, led to two important questions: *Which of the two genotypes is a better donor for cold-sensitive tropical indica cultivars? Is cold tolerance in IRGC100896 and Nipponbare due to conserved genetic mechanisms*, *or is it a similar outcome of distinct genetic mechanisms?* It is important to consider a previously established theory that phenotype alone may not always be an adequate indicator of the true genetic potential of a donor in breeding^25–28^. The possibility exists that the underlying genetic mechanisms may be distinct between two individuals without an obvious phenotypic contrast like in the case of the cold tolerance of *O*. *sativa* and *O*. *officinalis*. Complementation and epistasis may create novel effects when the positive attributes of each parent are combined by recombination.

This study represents the very first in-depth examination of the cold stress genetic mechanism of *O*. *officinalis*, and its uniqueness relative to the mechanism in *O*. *sativa* ssp. japonica. Because an annotated CC-genome reference is still underway, the IRGC100896 transcriptome was examined in this study with specific focus on genes with clear orthologs in Nipponbare^21,22^. Several major trends became apparent. First, transcriptome signatures showed similar trends in primary metabolic pathways and primary defenses such as radical scavenging and osmotic adjustment. In other words, no drastic differences in transcriptome signatures were evident to support major physiological differences between IRGC100896 and Nipponbare. It appeared that the baseline mechanisms for physiological adjustments and defenses are somehow conserved across the two representatives from the AA and CC genome groups.

Second, gene expression associated with most hormone biosynthetic pathways were also not significantly different between IRGC100896 and Nipponbare. The notion that ABA-regulatory mechanisms could fully explain stress tolerance variation across genotypes above and beyond the baseline defenses is questionable^17^. Similar patterns in ABA biosynthetic genes across IRGC100896 and Nipponbare were mirrored by similar ABA transcriptional networks as defined by the patterns in *ABRE* enrichment and expression of cognate activators *ABFs/bZIP/ABI4*. This was also echoed by the conserved patterns for ABA-independent regulon involving the *CRT/DRE-DREB* and *as1/ocs-bZIP/TGA* across IRGC100896 and Nipponbare^20,21,29^. ABA-mediated regulon along with ABA-independent (*CRT*/*DRE*-*DREB*) and oxidative-mediated (*as1/ocs-bZIP/TGA*) regulons are clearly shared features of the genetic mechanisms of IRGC100896 and Nipponbare.

Third, BL and SA biosynthetic pathways were uniquely upregulated in IRGC100896. BL-mediated network and its possible cross-talk with SA-signaling appeared to be an important feature of *O*. *officinalis* mechanism that was extensively fragmented in *O*. *sativa* ssp. japonica. BL is primarily involved in vascular bundle development, photomorphogenesis, stem and leaf angle and elongation, tillering, and germination^30,31^. It is also implicated with the maintenance of photosynthesis through the enhancement of PSII efficiency, intercellular CO_2_ concentration, stomatal regulation, antioxidant defense system, and osmotic adjustment^32–35^.

Perception of BL by *BRI1* protein leads to the accumulation of dephosphorylated *BES1* transcription factor in the nucleus, and consequent transcriptional changes facilitated by *BES1* binding to *BRRE* or *E-box* elements in target genes^36–38^. *BES1* activates *DREB*, *WRKY*, *NAC*, and *GRAS* transcription factors that control different sub-regulons implicated with stomatal regulation, antioxidant defense, osmotic adjustment, intracellular CO_2_ maintenance, and photosynthetic efficiency^39–42^. In IRGC100896 but not in Nipponbare, *BES1*-network genes were tightly co-upregulated with other major regulators known to be downstream to *BES1* (*i*.*e*., *DREB*, *WRKY*, *NAC*, *GRAS*). These genes have important roles in maintaining photosynthesis, radical scavenging, and osmotic adjustment.

Downstream targets of SA-mediated defenses in rice include *OsNPR1* and its interacting partners *OsWRKY45* and *OsTGA2/5/6*, all of which were prominent in the BL-mediated network of IRGC100896. BL modulates these transcription factors through an unknown regulator, while C_2_H_4_ has been suggested to antagonize the effects of SA and BL on these transcription factors^43,44^. These connections further strengthen the biological significance of the coupling of BL and SA biosynthesis in IRGC100896.

Earlier studies in Nipponbare showed that the mechanisms associated with early defenses to cold involve three sequentially expressing regulons^17,20,21,45^. First is the oxidative-mediated regulon involving *bZIP*-TGA-type transcription factors binding to the *as1/ocs-like* cis-elements. Second is ABA-dependent regulon that involves ABF, *bZIP* or *Myb* transcription factors, controlling their target genes through the *ABRE* cis-elements. Third is ABA-independent regulon that involves *DREB*/*CBF* transcription factors, controlling their target genes through the *CRT*/*DRE* cis-elements. These regulons contribute cumulatively to physiological adjustments and defenses through the enhancement of radical scavenging, repair of oxidative injuries, stomatal regulation, and osmotic adjustment. In IRGC100896, these processes appeared to be controlled by *BES1* through a different set of regulators and effectors.

Analysis of the larger *BES1* network (140 genes) indicated its extensive overlap with oxidative-mediated, ABA-dependent, and *DREB*/*CBF*-mediated regulons, all of which were active in both IRG1008096 and Nipponbare. This implies that in IRGC100896, the mechanisms for radical defense, stomatal regulation and osmotic adjustment are likely due to the synergy of all three regulatory pathways. In Nipponbare, similar physiological mechanisms appeared to be due mainly to oxidative-mediated, ABA-dependent, and *DREB*/*CBF*-mediated networks without *BES1* mechanism, hence *BES1* may be providing an extra layer of control for integrating defenses with growth in *O*. *officinalis*. This novel fine-tuning mechanism appeared fragmented in *O*. *sativa* ssp. japonica, perhaps as a consequence of domestication. *BES1-*network may also be an offshoot of a prominent role of BL in regulating growth under extreme environments where *O*. *officinalis* is well adapted.

Consistent with the comparable cold tolerance of *O*. *officinalis* and *O*. *sativa* ssp. japonica, the dominant functional signatures (*protection of photosynthesis*, *maintenance of intracellular CO*_*2*_, *oxidative defenses*, *osmotic adjustment*) implied by the transcriptome data were also remarkably similar across the two species. While the oxidative-mediated, ABA-dependent, and *DREB*-mediated regulons appeared to be generally conserved, *O*. *officinalis* has a functional *BES1* network for fine-tuning the integration of growth and stress-related responses. The fact that *O*. *officinalis* has an added regulatory feature that no longer exist in cultivars (*i*.*e*., *BES1)* reiterates its enormous value for expanding the genetic base of cultivars by wide hybridization.

In this study, we were also inspired by common observations that transgressive segregants are quite common in the progenies of crosses between cultivars and certain wild *Oryza*. These observations suggested that physiological complementation is possible between genetically distant genotypes although they may not exhibit clear phenotypic contrasts. We pursued our studies under the assumption that while *O*. *officinalis* and *O*. *sativa* ssp. *japonica* had similar cold tolerance, each may confer distinct molecular mechanisms with potential for positive complementation^46^. Indeed, the *BES1-*network that appeared to have been lost in cultivars provide a relevant proof of concept to test such hypothesis.

## Methods

### Plant materials, stress experiments, and RNA sampling

To substantiate the stress tolerance classification of Oryza species based on the bioclimatic models of Atwell *et al*.^4^, comparative physiological studies under cold stress were performed across a panel of accessions that included *O*. *sativa* ssp. japonica (cv. Nipponbare) as reference for AA-genome, *O*. *officinalis* IRGC100896 as reference for CC-genome, mega-variety IR64 (*O*. *sativa ssp*. *indica*) as sensitive check, and other representative diploid and tetraploid wild species (*O*. *rufipogon*/IRGC106424, *O*. *longistaminata*/IRGC110404, *punctata*/IRGC105690, *O*. *rhizomatis*/IRGC105442, *O*. *australiensis*/IRGC100882, *O*. *brachyantha*/IRGC101232, *O*. *eichingeri*/IRGC101424). Plants were established under control conditions at IRRI phytotron (25 °C to 28 °C day/22 °C to 24 °C night; 14 hr photoperiod) from germination to four-leaf stage (V_4_) in Yoshida hydroponics medium^18^. Cold stress experiments were performed in growth chamber at 4 °C day/night, 14 hr photoperiod, with tissue sampling for RNA extraction and physiological analysis at 24, 48, 72, and 168 hr. Electrolyte leakage analysis was performed after 48 hr of cold stress^22,47^. Post-stress plant recovery was assessed after 15 days at control conditions with three replicates. Plant recovery was assessed based on IRRI’s *Standard Evaluation Score (SES)* in a scale of 0 to 10 representing death to 100% recovery.

### RNA-Seq library construction and sequencing

Total RNA was extracted from frozen leaf tissues with the mirVana RNA kit (Ambion-ThermoFisher Scientific, USA). Samples from each time-point (0, 24, 48, 72,168 hr at 4 °C) were used to construct the time-course RNA-Seq libraries at 150 bp paired-end using the Illumina TruSeq RNA Library Prep kit V2 with three biological replicates (Illumina Inc., USA). Indexed libraries were sequenced across ten lanes on Illumina HiSeq2500, with sequencing coverage of ~90x for Nipponbare libraries, and ~60x for IRGC100896/W0065 libraries, each with ten technical replicates.

### Transcriptome data processing, assembly, and analysis

Sequence output from indexed RNA-Seq libraries were preprocessed with Cutadapt^48^, and mapped against the Nipponbare IRGSP1.0 reference for AA-genome (http://rapdb.dna.affrc.go.jp/download/irgsp1.html), GFF gene models, and *O*. *officinalis-W0002* reference for CC-genome by TopHat2 and Cufflinks^49,50^. The draft assembly of *W0002* genome (unpublished) was provided by Dr. Nori Kurata, National Institute of Genetics, Japan. Merging of sequence assemblies and test for statistical significance of mapped RNA-Seq reads were performed with Cuffdiff with default parameters (p-value = 0.05, FDR 5%).

The degree of orthologous sequence conservation is well established in the genus *Oryza*^5^. Within-genus orthologous transcripts (*japonica* versus *officinalis*) were established by searching the *W0002* draft for open reading frames and using them to establish gene models by Augustus (3.2.3)^51^. Using blat at default parameters, orthologous transcripts established from *officinalis* were aligned to Nipponbare *IRGSP1*.*0* gene models. The longest alignment was determined for each *O*. *officinalis* locus and final transcript sequences were annotated according to homologous loci in Nipponbare^52^. In cases where multiple *O*. *officinalis* loci corresponded to a single locus in Nipponbare, loci with the most stable mapping was used. Establishment of orthologous gene pairs for subsequent genome-wide comparison of gene expression and cis-element analysis were guided by established computational logic^5,53^.

### Analysis of differentially expressed genes and K-means clustering

For transcription factor transcripts, maximum fold-induction were calculated based on cumulative expression for each family in order to assess saturation. Gene-by-gene expression analysis was performed to identify the specific transcription factor transcripts with significant changes in expression in either or both IRGC100896 and Nipponbare. Analysis of expression of non-transcription factor transcripts was performed by first identifying the genes according to functional annotation using the RiceXPro database^54^. Threshold for differential expression was set at >2 (log_2_ scale) and p-value <0.01. Patterns of temporal co-expression was established across IRGC100896 and Nipponbare transcriptomes by K-means clustering in R-package MBCluster version 3.3.3 using the negative binominal with eight total clusters^55^.

### Metabolic and hormone biosynthetic pathway mapping

Transcripts relevant to metabolic and hormone biosynthetic pathways were first identified from transcriptome datasets and their abundances were compared across the two genotypes. Transcripts were mapped to the reference metabolic pathways using the KaPPA-View analysis for glycolysis, TCA cycle, starch synthesis and catabolism, triacylglyceride synthesis and catabolism, photosynthesis (chlorophyll biosynthesis, Calvin cycle), ubiquinone synthesis, ROS scavenging (catalase, peroxidase, superoxide dismutase, GSH synthesis), osmotic adjustment (trehalose, sucrose, and proline) and hormone biosynthesis (http://kpv.kazusa.or.jp)^56^.

### Cis-element analysis

Genomic sequences corresponding to −3,000 to +2,000 regions were extracted for all loci with mapped transcripts in both *W0002* and Nipponbare. These sequences were scanned for motifs corresponding to known and/or putative cis-elements by MAMA on CUDA version 7.5^57^. Of all the motifs occurring along the −3,000 to +2,000 region, those that were specifically enriched around −500 region or −150 to +50 interval only among upregulated genes were given high MAMA scores. High MAMA-scoring motifs were used to generate spatial maps of −1,200 to +500 regions of all candidate genes for biological hypothesis testing. Cis-element spatial maps were visualized using the Matlab version R2017a (http://www.mathworks.com). Baseline annotation of all putative cis-elements in PLACE database^58^ was further elaborated with additional information from the literature^29,45^.

### Transcriptional network modeling

The dataset used for modeling the brassinosteroid (BL) network in IRGC100896 included a subset of tightly co-expressed genes with either high (8 to 10 copies) or very high (at least 11 copies) density of sequence motifs for *brassinosteroid response element* (*BRRE*) across the −1,200 to +500 regions. Nipponbare loci orthologous to the *BRRE*-enriched and co-regulated genes in IRGC100896 were used to establish the corresponding networks for *O*. *sativa* ssp. *japonica*. Models of co-expression networks were visualized using Cytoscape version 3.5.1 (http://www.cytoscape.org/^59^. In all network models, length of the edge reflects the strength of co-expression.

## Electronic supplementary material

Supplementary Tables and Figures

## Data Availability

The RNA-Seq data described in this manuscript are publicly available as DDBJ accession DRA006704. Supplementary data files 1–21 accompany this paper.
