# Supplementary material for: Potential of Oryza officinalis to augment the cold tolerance genetic mechanisms of Oryza sativa by network complementation
Source: Sci Rep. 2018 Nov 5;8:16346. doi: 10.1038/s41598-018-34608-z (PMC6218501; doi:10.1038/s41598-018-34608-z)
Supplement: Supplementary file 1 — Supplementary Tables and Figures [file 41598_2018_34608_MOESM1_ESM.docx]

**Potential of the *Oryza officinalis* to augment the cold tolerance genetic mechanisms of *Oryza sativa* by network complementation**

Ai Kitazumi^1^, Isaiah C. M. Pabuayon^1^, Hajime Ohyanagi^2,3^, Masahiro Fujita^2^,

Bipush Osti^1^, Matthew Shenton^2,4^, Yusuke Kakei^5^, Yasukazu Nakamura^6^,

Darshan S. Brar^7^, Nori Kurata^2^, Benildo G. de los Reyes^1*^

1. Department of Plant and Soil Science, 219 Experimental Sciences Building, Texas Tech University, Lubbock, TX 79409 USA; **Corresponding author*
2. Plant Genetics Laboratory, National Institute of Genetics, Mishima, Shizuoka 411-8540, Japan
3. Computational Bioscience Research Center, Biological and Environmental Sciences and Engineering Division, King Abdullah University of Science and Technology, Thuwal, 23955-6900, Saudi Arabia
4. School of Agriculture, Meiji University, 1-1-1 Higashi-Mita, Tama-ku, Kawasaki-shi, Kanagawa 214-8571, Japan
5. Institute of Vegetable and Floriculture Science, National Agriculture and Food Research Organization, Mie, 514-2392, Japan
6. Genome Informatics Laboratory, National Institute of Genetics, 1111 Yata, Mishima, Shizuoka, 411-8540, Japan
7. Plant Breeding, Genetics and Biotechnology Division, International Rice Research Institute, Los Banos, Philippines

***Supplementary Figure S1.*** Differences in plant recovery among representative Oryza accessions after exposure to cold stress. Osj = *sativa-japonica*, Nipponbare; Osi = *sativa-indica*, IR64; Or = *rufipogon*; Ol = *longistaminata*; Oo = *officinalis*; Oe = *O. eichingeri*; Or = *rhizomatis*; Oa = *australiensis*; Ob = *brachyantha*; Op = *punctata*.

***Supplementary Table S2.*** Summary and mapping statistics of the RNA-Seq libraries of IRGC100896 and Nipponbare.

| **Species** | **Treatment** | **Left (million reads)** | **Right (million reads)** | **Map rates (%)** |
| --- | --- | --- | --- | --- |
| *Oryza sativa* ssp. *japonica* cv. Nipponbare | Control (0H) | 116.612586 | 116.612586 | 92.38 |
|  | 24 hours in 4°C | 128.808263 | 128.808263 | 93.01 |
|  | 48 hours in 4°C | 119.322768 | 119.322768 | 91.78 |
|  | 72 hours in 4°C | 133.127231 | 133.127231 | 92.09 |
|  | 168 hours in 4°C | 115.159482 | 115.159482 | 92.21 |
| *Oryza officinalis* (IRGC100896) | Control (0H) | 127.845591 | 127.845591 | 82.14 |
|  | 24 hours in 4°C | 115.268543 | 115.268543 | 82.05 |
|  | 48 hours in 4°C | 114.912724 | 114.912724 | 82 |
|  | 72 hours in 4°C | 117.252454 | 117.252454 | 80.98 |
|  | 168 hours in 4°C | 118.638868 | 118.638868 | 55.44 |

***Supplementary Figure S3*.** Identification of orthologous and non-orthologous transcripts and gene loci across the IRGC100896 and Nipponbare RNA-Seq datasets based on IRGSP 1.0 reference for *Oryza sativa* L. var. Nipponbare.

***Supplementary Figure S4*.** KaPPA-view pathway and transcript abundance map of the glycolytic pathway across IRGC100896 (*purple*) and Nipponbare (*green*). The rate-limiting step is highlighted by the grey box.

***Supplementary Figure S5*.** KaPPA-view pathway and transcript abundance map of the TCA cycle across IRGC100896 (*purple*) and Nipponbare (*green*). The rate-limiting step is highlighted by the grey box.

***Supplementary Figure S6*.** KaPPA-View pathway and transcript abundance map of the starch metabolic pathway across IRGC100896 (*purple*) and Nipponbare (*green*). The rate-limiting step is highlighted by the grey box.

***Supplementary Figure S7*.** KaPPA-View pathway and transcript abundance map of the triacylglyceride biosynthetic pathway across IRGC100896 (*purple*) and Nipponbare (*green*). The rate-limiting step is highlighted by the grey box.

***Supplementary Figure S8*.** KaPPA-View pathway and transcript abundance map of the ubiquinone biosynthetic pathway across IRGC100896 (*purple*) and Nipponbare (*green*). The rate-limiting step is highlighted by the grey box.

***Supplementary Figure S9.*** KaPPA-View pathway and transcript abundance map of chlorophyll biosynthesis showing the relative pathway activities in IRGC100896 (*purple*) and Nipponbare (*green*). The rate-limiting step is indicated by the grey box. Abbreviated enzymes and compounds are: GSA = *glutamate-1-semialdehyde*; PBG = *porphobilinogen*; PBGD = *porphobilinogen deaminase*; UROD = *Uroporphyrinogen III decarboxylase*; Proto IX = *protoporphyrinogen IX*; PPO = *protoporphyrinogen oxidase*; MgP = *Mg-protoporphyrin monomethylester*; MgPMe = *Mg-protoporphyrin monomethylester*; MgP(IX)MT = *Mg-protoporphyrin IX methyltransferase*

***Supplementary Figure S10.*** KaPPA-View pathway and transcript abundance map of Calvin cycle showing the relative pathway activities in IRGC100896 (*purple*) and Nipponbare (*green*). The rate-limiting step is indicated by the grey box. Abbreviated enzymes and compounds are: RuBP = *Ribulose 1,5-bisphosphate*; 1,3-BPG = *1,3-bisphosphoglycerate*; PPK = *Phosphoglycerate kinase*; 3-PG = *3-phosphoglycerate*; GAPDH = *Glyceraldehyde-3-phosphate dehydrogenase*; GAP = *Glyceraldehyde-3-phosphate*; TPI = *Triose-phosphate isomerase*; DHAP = *Dihydroxyacetone phosphate*; SH17BP = *D-Sedoheptulose-1,7-bisphosphate*; SBPase = *sedoheptulose bisphosphatase*; SH7P = *D-Sedoheptulose-7-phosphate*; RPI = *Ribose-5-phosphate isomerase*; Ru5P = *ribulose-5-phosphate*; RPE = *ribulose-5-phosphate epimerase*.

***Supplementary Figure S11.*** KaPPA-View pathway and transcript abundance maps for trehalose biosynthesis with the rate-limiting steps highlighted by the grey box.

***Supplementary Figure S12.*** KaPPA-View pathway and transcript abundance maps for sucrose biosynthesis with the rate-limiting steps highlighted by the grey box.

***Supplementary Figure S13.*** KaPPA-View pathway and transcript abundance map of the gibberellic acid (GA) biosynthetic pathway across IRGC100896 (*purple*) and Nipponbare (*green*). The rate-limiting step is highlighted by the grey box.

***Supplementary Figure S14*.** KaPPA-View pathway and transcript abundance map of the jasmonic acid (JA) biosynthetic pathway across IRGC100896 (*purple*) and Nipponbare (*green*). The rate-limiting step is highlighted by the grey box.

***Supplementary Figure S15*.** KaPPA-View pathway and transcript abundance map of the cytokinin (ZT) biosynthetic pathway across IRGC100896 (*purple*) and Nipponbare (*green*). The rate-limiting step is highlighted by the grey box.

***Supplementary Figure S16*.** Full KaPPA-View pathway and transcript abundance map of the ethylene (C_2_H_4_) biosynthetic pathway across IRGC100896 (*purple*) and Nipponbare (*green*). The rate-limiting step is highlighted by the grey box.

***Supplementary Figure S17*.** KaPPA-View pathway and transcript abundance map of the auxin (IAA) biosynthetic pathway across IRGC100896 (*purple*) and Nipponbare (*green*). The rate-limiting step is highlighted by the grey box.

***Supplementary Table S18*.** List of cis-elements that are most highly enriched among the cold co-upregulated gene clusters across IRGC100896 and Nipponbare.

***Supplementary Figure S19*.** Heat maps showing the patterns of expression of all transcription factor families across the IRGC100896 and Nipponbare RNA-Seq datasets. Heat maps were based on unscaled expression values.

***Supplementary Figure S20*.** Selection of the *BES1* transcriptional network components across the IRGC100896 and Nipponbare RNA-Seq datasets. A subset of transcription factor and non-transcription factor transcripts with stable induction under cold stress was established first from the IRGC100896 RNA-Seq dataset (n = 808). These transcripts represented two large expression clusters defined as: *late-induced by cold* or *sustained induced by cold*. *BES1* transcription factor, the key component of BL-mediated transcription was among those included in the *sustained induction by cold* group. Genomic loci for these transcripts were extracted from the CC-genome draft assembly (National Institute of Genetics, Japan) and investigated for the enrichment of *BRRE* and *BRRE*-like sequence motifs across the -1,200 to +200 regions. Loci encoding this subset of cold-induced transcripts were further placed into two distinct subgroups with either high or very high enrichment of *BRRE*-related sequence motifs. Orthologous transcripts from the Nipponbare RNA-Seq dataset formed four expression clusters defined as: *not induced by cold or NI*, *early induced by cold*, *late induced by cold*, or *sustained induced by cold*. *BES1* transcription factor belong to the *NI* group. Orthologous transcripts selected from the Nipponbare were also significantly depleted of *BRRE* and/or *BRRE*-like sequence motifs across the upstream regulatory regions (-1,200 to +200) of their genomic loci. These trends were indicative of the fragmentation of the network components in Nipponbare relative to IRGC100896.

***Supplementary Table S21*.** Table listing all the candidate BES1-network genes included in network modeling. Cis-element data was based on -1,200 to +200.
